# Are there candidates for high-dose chemotherapy in ovarian carcinoma?

**DOI:** 10.1186/1756-9966-31-87

**Published:** 2012-10-16

**Authors:** Renaud Sabatier, Anthony Gonçalves, François Bertucci, Maria-Antonietta Capiello, Frédérique Rousseau, Eric Lambaudie, Christian Chabannon, Patrice Viens, Jean-Marc Extra

**Affiliations:** 1 Department of Medical Oncology, Institut Paoli-Calmettes, 232 Bd Ste-Marguerite, Marseille 13273, France; 2 Department of Molecular Oncology, Centre de Recherche en Cancérologie de Marseille, UMR1068 INSERM, Marseille, France; 3 Department of Molecular Pharmacology, Centre de Recherche en Cancérologie de Marseille, UMR1068 INSERM, Marseille, France; 4 UFR Medicine, University of Mediterranean, Marseille, France; 5 Department of Surgical Oncology, Institut Paoli-Calmettes, Marseille, France; 6 Department of Hematology, Institut Paoli-Calmettes, Marseille, France

**Keywords:** Ovarian carcinomas, Prognosis, High dose chemotherapy, Stem cell support

## Abstract

**Background:**

Prognosis of advanced ovarian carcinomas (AOC) remains poor with a 5-year survival of 30%. Benefit from high-dose chemotherapy (HDC) in this disease has not been demonstrated to date.

**Methods:**

To evaluate the value of HDC as consolidation treatment after surgery and platinum/taxane-based therapy, we designed a monocentric retrospective comparative study. We used a subset approach to identify parameters associated with HDC efficacy.

**Results:**

One hundred and three AOC patients treated with conventional chemotherapy alone (CCA) were compared to 60 patients receiving HDC plus hematopoietic stem cell support. After a median follow-up of 47.5 months there was no overall survival (OS) advantage for the HDC group in the whole population (*p*=0.29). Nevertheless, HDC was associated to a better outcome in young patients (≤50 years), both in term of progression-free survival (*p*=0.02, log-rank test) and OS (*p*=0.05, log-rank test). Median OS was 54.6 and 36 months in the HDC and CCA groups, respectively.

**Conclusions:**

Although randomized trials failed to demonstrate any benefit for HDC in AOC patients, this study suggests that young patients may derive a substantial advantage from receiving it after the standard treatment. Further prospective studies are warranted to confirm this gain and to search for the biological processes associated with this improvement.

## Background

Ovarian carcinoma is the first cause of death by gynecologic malignancy in western countries. In 2010 in USA, around 22 000 cases were diagnosed and 14 000 deaths were reported [1]. Such a poor prognosis is due to late diagnosis and relative lack of efficacy of current treatments. The therapeutic sequence used by most of clinicians is maximal cytoreductive surgery (also called debulking surgery) followed by adjuvant chemotherapy for undifferentiated or advanced tumors [2-7]. Nevertheless, 20% of patients are initially refractory to this treatment and more than 50% of patients who are initially in complete remission will relapse and ultimately succumb from disease [8,9]. Consequently, overall survival is quite reduced and has remained stable since 20 years (30-40% at five years for all stages). Early stages have a favorable prognosis (~90%), while life expectancy is only 30% after 5 years when disease is extended to peritoneal cavity and only 5-10% when there is distant metastasis [8,9].

A combination of a platinum agent and paclitaxel is the standard therapy with benefits in terms of response, progression-free and overall survivals, leading in stages III and IV to a median survival of more than 35 months [10,11]. Several laboratory models [12] as well as retrospective analyses of clinical studies [13,14] have strongly suggested that chemotherapy dose could favorably influence ovarian cancer outcome. Major chemotherapy dose intensification using alkylating agents with autologous hematopoietic stem cell support (HSCS) has been investigated in this setting, with encouraging results in pilot studies [15-18]. However, these promising results have not been confirmed in randomized phase III trials [19,20], and high-dose chemotherapy (HDC) is currently not recommended for advanced ovarian carcinomas (AOC).

Nevertheless, ovarian cancer is clinically heterogeneous. Patients with morphologically similar, advanced-stage tumors display a broad range of clinical outcomes. Features currently used for prognosis and chemotherapy decision are clinicopathological and include patient’s age, performance status, FIGO stage, histological tumor grade and subtype, initial surgery results and response to chemotherapy. These factors were not incorporated in the initial design of randomized studies although they might be associated with different responses to HDC.

The present study is a retrospective comparative survival analysis, including subsets analysis based on usual clinicopathological features. A survival comparison was done between 103 patients with AOC treated by surgery plus platinum/taxane-based conventional chemotherapy alone (CCA) and 60 patients who received the same treatment plus HDC and autologous HSCS.

## Methods

### Population description

Patients were selected in our institutional “Ovarian Cancer” database, which included all ovarian cancer patients treated at the Institut Paoli-Calmettes (Marseilles, France) since 1995. Eligible patients were aged between 18 and 64 years and had histologically proven invasive ovarian carcinoma with advanced (FIGO stage IIIc) or metastatic (FIGO stage IV) disease at diagnosis.

All patients were treated using a standard multimodal approach including surgery and platinum/taxane-based chemotherapy. In the “HDC” group, patients also received HDC with HSCS. Hematological rescue consisted of autologous hematopoietic stem cells collected from peripheral blood.

After completion of treatment, patients were evaluated at 3-month intervals for the first 2 years and at 6-month intervals thereafter. Evaluations included clinical examination and blood tests with CA125 assessment. CT scan evaluations were performed every 6 months for the first 5 years and yearly thereafter. Other examinations were performed only when indicated.

The study was approved by our institutional review board. According to the French law, since it was a retrospective study without biological research and without therapy modification, no personal consent was required.

### Statistical analysis

Differences in patient characteristics between the two chemotherapy groups (with *vs.* without HDC) were tested by the Fisher’s exact test (categorical variables) or the Student’s *t*-test (continuous variables). Tested parameters were age at diagnosis (with a threshold at 50 years old), performance status, FIGO stage, histological subtype (serous *vs.* others), histological grade according to Silverberg classification (grade 1 and 2 were pooled), presence of residual disease after surgery, presence of a clinical remission after platinum/taxane-based therapy (according to clinical and radiological examinations), CA125 normalization after platinum/taxane-based therapy.

Progression-free survival (PFS) was calculated from the date of diagnosis until date of first disease progression. Overall survival (OS) was calculated from the date of diagnosis until date of death of any cause. Follow-up was measured from the date of diagnosis to the date of last news for live patients. Data concerning patients without disease progression or death at last follow-up were censored. Survival curves were estimated using the Kaplan-Meier method, and compared with the log-rank test. The prognostic impact of above-cited factors and chemotherapy regimen was assessed by the Cox regression method both in univariate and multivariate analysis. Multivariate analyses only included variables with p-value lower than 5% in univariate analysis. All statistical tests were two-sided at the 5% level of significance. Statistical analyses were performed using SPSS software (version 16.0).

## Results

### Patients and treatment

One hundred sixty-three patients with advanced ovarian carcinomas treated at our institution between April 1995 and July 2009 were included in this study. Tumor characteristics are listed in Table 1. Median age at diagnosis was 54 years (standard deviation, 8.7 years) and 68% were older than 50 years. Fifty three percent were grade II serous tumors. Complete cytoreductive surgery could not be achieved for 41% of patients. Seventy percent presented no clinical residual disease after conventional treatment including surgery and chemotherapy. All patients received a platinum/taxane-based chemotherapy. Ninety percent of patients received carboplatin, 10% cisplatin, 79% paclitaxel and 21% docetaxel. Carboplatin was given every three weeks, according to the Calvert’s formula with an area under curve of 6 before and 5 after January 2005. Cisplatin was given every three weeks at a dose of 75 mg/m^2^. Paclitaxel was administered every three weeks at the dose of 175 mg/m^2^ until 2008, and then weekly at the dose of 80 mg/m^2^. Docetaxel was given with a 3-weeks frequency, at the dose of 75 mg/m^2^. Patients received a median of 6 cycles, with a minimum of 1, and a maximum of 8 cycles.

**Table 1:** Clinicopathological features of advanced ovarian carcinomas with and without high-dose chemotherapy

			**CCA**	**HDC**	**p -value**	**Odd or Hazard Ratio (95CI)**
	**N**		**N (%)**	**N (%)**		
			**103**	**60**		
Follow-up (median, months)	163		46.7	48.2	0.08***	
Median Age (years)	163		56,0	53,0 0	09***	
Age	163				0.73****	1.15 [0.55-2.45]
		≤50y	34 (33)	18 (30)		
		>50y	69 (67)	42 (70)		
OMS	117				0.17****	0.35 [0.06-1.37]
		0-1	63 (81)	36 (92)		
		2-3	15 (19)	3 (8)		
FIGO	163				0.33****	1.47 [0.63-3.39]
		IIIc	84 (82)	45 (75)		
		IV	19 (18)	15 (25)		
Histological subtype	163				0.62****	0.82 [0.40-1.65]
		Serous	62 (60)	39 (65)		
		Others	41 (40)	21 (35)		
Grade	98				0.01****	0.32 [0.12-0.81]
		1-2	19 (31)	21 (58)		
		3	43 (69)	15 (42)		
Cytoreductive surgery	160					
		Complete	56 (56)	40 (67)	0.24****	0.64 [0.31-1.30]
		residual disease	44 (44)	20 (33)		
Clinical complete response*	161					
		Yes	63 (62)	50 (83)	0.007****	0.33 [0.14-0.77]
		No	38 (38)	10 (17)		
CA-125**	149				0.66****	0.75 [0.27-1.92]
		Normal	73 (80)	49 (85)		
		>Normal	18 (20)	9 (15)		
Time from end of initial CT to HDCT (median, months)	61		NA	2.8	NA	NA
Median PFS (months)			18.1	20.1	0.09*****	
Median OS (months)			41.3	47.3	0.24*****	

*CCA*, conventional chemotherapy alone; *HDC*, high-dose chemotherapy; *N*, number of cases with data available; *95CI*, 95% confidence interval; *OMS*, performance status; *NA*, not asssessable; *PFS*, progression-free survival; *OS*, overall survival. *Clinical and radiological complete response after platinum and taxane-based chemotherapy; **, CA-125 rate after platinum and taxane-based chemotherapy; ***, *T*-test; ****, Fisher's exact test; *****, Log-rank test.

**Figure 1:**
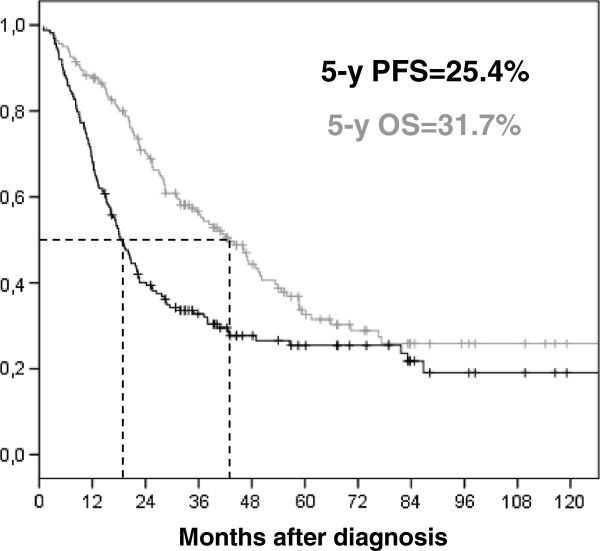
**Survival curves of the whole population (n=163).** Progression-free survival in black (median PFS = 18.8 months), and Overall survival in grey (median OS = 42.7 months), + censored data.

Seventy-one patients underwent second look surgery after platinum/taxane-based chemotherapy. Of them, 25 presented a pathological complete response. Eighteen percent did not reach CA125 normalization after standard treatment achievement. Median PFS of the whole population was 18.8 months, with a 5-year PFS of 25.4%. Median OS was 42.7 months, with a 5-year OS of 32.6% (Figure 1).

Out of these 163 patients, two groups were distinguished with respect to the regimen of chemotherapy: 103 patients (63%) received conventional chemotherapy alone (“CCA group”) and 60 patients (37%) received HDC with HSCS after completion of a platinum/taxane-based regimen (“HDC group”). Median time from platinum/taxane-based chemotherapy completion to HDC was 2.8 months. Because of the large period of inclusion, HDC regimens were heterogeneous. Nevertheless, all patients received alkylating agents. The details of the HDC regimen are noted in Table 2. Median and mean numbers of re-injected hematopoietic stem cells (CD34 positive cells) per patient were 6.1 million and 8.3 million per Kg, respectively.

**Table 2:** High dose chemotherapy regimen in the high-dose chemotherapy group (N=60)

	**N (%)**
Carboplatin AUC 18	12 (20)
Cyclophosphamide 60mg/kg/d (d-3 to d-2) + melphalan 140 mg/m^2^ d-1	32 (53)
Cycle 1: cyclophosphamide 60mg/kg/d (d-3 to d-2) + melphalan 140 mg/m² d-1 +	
Cycle 2: thiotepa 300mg/m²/d d-3 to d-2	8 (13)
Melphalan 140 mg/m² d-1	3 (5)
Thiotepa 300mg/m²/d d-3 to d-2	1 (2)
Cycle 1: melphalan 140 mg/m² d-1 + Cycle 2: thiotepa 300mg/m²/d d-3 to d-2	2 (3)
Topotecan 7,5mg/m²/d (d-6 to d-2)	2 (3)*

*N*, number of patients; *AUC*, area under curve; *d*, day; *, patients treated in the ITOV 01 trial.

There was no statistically significant difference between the two subsets (Table 1), except for clinical complete remission after platinum/taxane-based regimen: 62% in the CCA group versus 83% in the HDC group (*p*=7.0 E-03, Fisher’s exact test). Such an imbalance can be explained by the fact that only patients with complete or at least partial response were candidate to HDC. It is of note that no toxic death was observed in the HDC arm.

### Pathological response

Seventy-one patients underwent second look surgery (SLS) at the end of the platinum/taxane-based treatment. Among them, 27 received HDC after SLS. There was no statistical difference in pathological response between the HDC and the CCA subsets: seven pathological complete responses were observed in the HDC subset (26%) and eighteen in the CCA group (41%), *p*=0.31 (Fisher’s exact test).

### Outcome and survival

Median follow-up was 47.5 months. There were 79 disease progressions and 64 deaths in the conventional therapy group versus 40 and 35, respectively in the HDC group. Outcome evaluation according to therapy showed that median PFS and OS were similar with 20.1 and 47.3 months in the HDC group versus 18.1 and 41.3 months in the CCA group, respectively.

### Prognostic parameters

**Figure 2:**
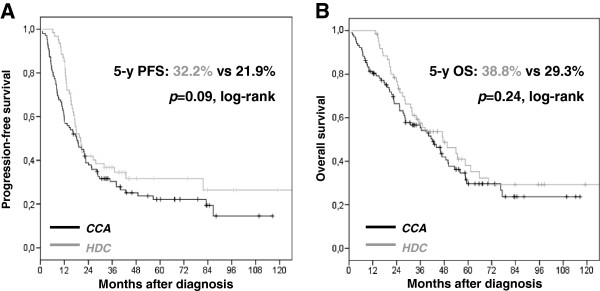
**Progression-Free Survival (A) and Overall Survival (B) according to chemotherapy regimen in the whole population.** Conventional chemotherapy alone (CCA) alone in black, n=103; conventional chemotherapy plus high-dose chemotherapy in grey, n=60, + censored data.

In the whole population (Table 3A), PFS was influenced by debulking surgery results (hazard ratio (HR) for progression of 0.38 if no residual disease was present), response to therapy (HR=0.33 in case of complete clinical response (CCR)), and CA125 normalization (HR=0.45). Outcome was not significantly improved when HDC was added (PFS, *p*=0.09; OS, *p*=0.24), (Figure 2). Multivariate analysis showed that only two features had an independent prognostic value in the whole population: surgical results and clinical response to initial chemotherapy.

**Table 3:** Prognostic parameters (PFS), Cox regression analysis

**A. Whole population**								
	**Univariate analysis**				**Multivariate analysis**			
	**N**	**HR**	**95CI**	**p -value**	**N**	**HR**	**95CI**	**p -value**
Age (>50y vs ≤50y)	163	1.12	0.76-1.66	0.57				
OMS (0-1 vs 2-3)	117	1.53	0.88-2.67	0.14				
FIGO (IIIc vs IV)	163	0.7	0.45-1.08	0.1				
Histology (serous vs others)	163	0.95	0.66-1.39	0.8				
Grade (1-2 vs 3)	98	1.2	0.93-1.55	0.16				
Serous grade 3 (vs others)	98	1.42	0.80-2.52	0.23				
Surgery (complete vs non complete)	160	0.38	0.26-0.54	2.23 E-07	147	0.57	0.37-0.87	0.01
Complete clinical remission (Yes vs No)	161	0.33	0.23-0.49	2.14 E-08	147	0.55	0.33-0.92	0.02
CA-125 (normal vs >normal)	149	0.45	0.29-0.71	6.9 E-04	147	0.77	0.45-1.32	0.34
Time from end of initial CT to HDC			NA					
Treatment (CCA vs HDC)	163	1.39	0.95-2.03	0.09				
**B. According to chemotheraphy regimen, univariate analysis**								
	**Conventional CT**				**High dose CT**			
	**N**	**HR**	**95CI**	**p -value**	**N**	**HR**	**95CI**	**p -value**
Age (>50y vs ≤50y)	103	0.83	0.52-1.33	0.44	60	2.03	0.96-4.29	0.06
OMS (0-1 vs 2-3)	78	1.56	0.84-2.89	0.16	39	0.96	0.22-4.17	0.95
FIGO (IIIc vs IV)	103	0.93	0.52-1.70	0.82	60	0.4	0.20-0.78	0.007
Histology (serous vs others)	103	1.24	0.78-1.97	0.37	60	0.83	0.44-1.58	0.56
Grade (1-2 vs 3)	62	1.17	0.85-1.61	0.35	36	1.08	0.67-1.72	0.76
Serous grade 3 (vs others)	62	0.81	0.57-1.15	0.24	36	0.98	0.51-1.87	0.94
Surgery (complete vs non complete)	100	0.29	0.18-0.46	2.2 E-07	60	0.65	0.34-1.22	0.18
Complete clinical remission (Yes vs No)	101	0.32	0.20-0.51	1.78 E-06	60	0.44	0.20-0.97	0.04
CA-125 (normal vs >normal)	91	0.32	0.18-0.56	6.41 E-05	58	1.21	0.53	2.74
Time from end of initial CT to HDC			NA		60	0.97	0.86-1.09	0.59
Treatment (CCA vs HDC)			NA				NA	

*PFS*, progression-free survival; *N*, number of cases with data available; *95CI*, 95% confidence interval; *HR*, hazard ratio; *OMS*, performance status; *HDC*, high-dose chemotherapy; *CCA*, conventional chemotherapy alone.

We then explored the prognostic value of the usual clinicopathological features in each treatment arm.

We first examined PFS. In the CCA group, PFS was influenced by debulking surgery results (HR=0.29), clinical response to therapy (HR=0.32), and CA125 normalization (HR=0.32). In the HDC arm, age (HR=2.07 if older than 50 years) FIGO stage (HR=0.41 for stage IIIc) and clinical response to initial treatment (HR=0.46) had a prognostic value (Table 3B). When focusing only in the pre-treatment clinicopathological features, only age and FIGO stage had a prognostic value in the HDC group.

**Figure 3:**
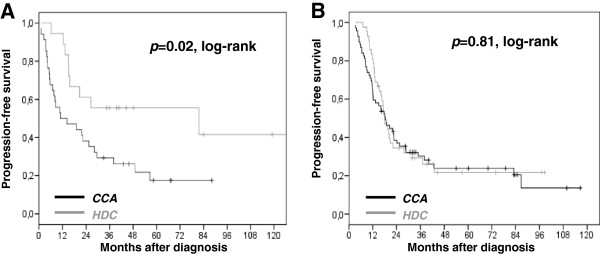
**Progression-Free Survival according to chemotherapy regimen.** Conventional chemotherapy alone (CCA) in black or plus high-dose chemotherapy (HDC) in grey. (**A**) In patients under 50 years of age (n=52), median PFS was 11 months in the CCA subset versus 81.7 months in the HDC subset. (**B**) In patients older than 50 years old (n=111), median PFS was 18.3 months in the CCA subset versus 17.9 months in the HDC subset. + censored data.

Impact of HDC on PFS according to these last two features was analyzed. HDC significantly improved PFS in young patients (*p*=0.02, log-rank test), but had no prognostic value in women older than 50 years (*p*=0.81, log-rank test), (Figure 3). In the same way, HDC increased PFS in stage IIIc patients (*p*=0.03, log-rank test), but not in stage IV cases (*p*=0.94, log-rank test).

Cox regression analyses performed in both young patients and stage IIIc cases found that PFS was significantly affected by HDC, surgical results, complete remission and Ca125 normalization after conventional treatment. Young patients had a 2.44-fold rate of progression if they did not receive HDC (Table 4); and stage IIIc patients a 1.61-fold rate of progression if they did not receive HDC (Additional file 1: Table S1). By multivariate analyses HDC had an independent prognostic value in young patients (Table 4), but not in stage IIIc cases (Additional file 1: Table S1).

**Table 4:** Prognostic features (PFS) in young patients (≤50 years), Cox regression analyses

	**Univariate analysis**				**Multivariate analysis**			
	**N**	**HR**	**95CI**	***p*-value**	**N**	**HR**	**95CI**	***p*-value**
OMS (0-1 vs 2-3)	36	1.76	0.71-4.38	0.22				
FIGO (IIIc vs IV)	52	0.57	0.25-1.33	0.19				
Histology (serous vs others)	52	0.81	0.51-1.56	0.52				
Grade (1-2 vs 3)	31	1.31	0.83-2.08	0.25				
Serous grade 3 (vs others)	31	1.06	0.59-1.88	0.85				
Surgery (complete vs non complete)	52	0.29	0.15-.058	4.97 E-07	51	0.43	0.19-0.94	0.034
Complete clinical remission (Yes vs No)	51	0.22	0.11-0.45	3.65 E-05	51	0.33	0.15-0.74	0.007
CA-125 (normal vs >normal)	44	1.87	0.84-4.16	0.12				
Treatment (CCA vs HDC)	52	2.44	1.14-5.25	0.02	51	2.31	1.06-5.04	0.036

*PFS*, progression-free survival; *N*, number of cases with data available; *95CI*, 95% confidence interval; *HR*, hazard ratio; *OMS*, performance status; *CCA*, conventional chemotherapy alone; *HDC*, high-dose chemotherapy.

**Figure 4:**
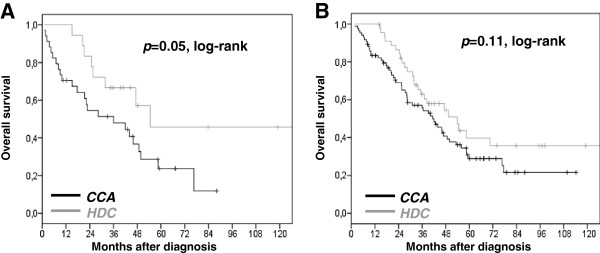
**Overall survival after conventional chemotherapy alone (black) or plus high dose chemotherapy (grey).** (**A**) In patients under 50 years of age (n=52) median OS was 36 months in the CCA subset versus 54.6 months in the HDC subset; (**B**) in stage IIIc cases (n=129) median OS was 42 months in the CCA subset versus 49.5 months in the HDC subset; + censored data.

We then explored the impact of chemotherapy regimen on OS according to the two factors independently associated with a PFS improvement induced by HDC (young age and FIGO stage IIIc). We could observe that HDC plus HSCS significantly improved survival only when age was under 50 years, but not in stage IIIc patients (Figure 4). Median overall survival was highly increased in young patients treated with HDC (54.6 months) when compared to conventional therapy alone (36 months), (*p*=0.05). Effect of HDC according to FIGO stage IIIc was less important and non significant: median OS was 53.9 months in the HDC subset versus 41.3 months in the CCA subset (*p*=0.11).

It is worth to note that the prognostic value of HDC was not modified by the initial response to treatment. HDC improved survival in young patients whatever the response to initial therapy was: median PFS was 5 months for CCA *vs.* 15 months for HDC in patients with residual disease after treatment; and 38 months for CCA whereas it had not been reached after a follow-up of 47 months in the HDC group for cases with initial CCR and CA-125 normalization.

## Discussion

Even though HDC plus HSCS cannot be considered as a standard of care for all AOC patients, results from this monocentric comparative retrospective study including 163 patients suggest that it may be beneficial to young patients. In women under 50 years of age, addition of HDC to platinum/taxane-based chemotherapy improves not only PFS (*p*=0.02), but also OS (median of 54.6 months versus 36 months with conventional therapy alone, *p*=0.05).

Despite advances in chemotherapy and multidisciplinary management of ovarian carcinomas, the prognosis of patients with advanced stages (FIGO III/IV) remains poor. Median PFS and OS of our cohort treated with a platinum/taxane combination alone (18.1 and 41.3 months, respectively) were similar to those of phase III pivotal studies: 18 and 38 months [10], and 19.4 and 48.7 months [6] with cisplatin and paclitaxel; 20.7 and 57.4 months for carboplatin and paclitaxel [6]. Our population was thus similar to previously described cohorts. Prognosis is known to be dramatically influenced by cytoreductive surgery and response to adjuvant platinum/taxane-based chemotherapy. However, even good responders to initial treatment often have a poor prognosis due to secondary relapse. Such relapses are generally chemoresistant and remain the major cause of death. Thus, it may be useful to treat chemosensitive patients in order to kill residual clones and avoid the chemoresistant relapse. Different consolidation therapies have been considered: conventional maintenance chemotherapy, intraperitoneal treatment with chemotherapy and/or hyperthermia, and HDC with HSCS. The latter has been widely used in the context of poor risk hematological malignancies and sometimes in chemosensitive solid tumors such as metastatic breast cancer [21-25] or germ cell tumors [26] with controversial results.

The main toxicity of high-dose alkylating agents is hematological. Stem cell transplantation is needed in such treatment strategies to limit the duration and consequences of aplasia. Nevertheless, severe infection can always occur during grade 4 neutropenia and remains the major potential risk during severe aplasia. However we observed no toxic death after HDC in this study.

Several promising but preliminary studies have reported that HDS plus HSCS may improve ovarian cancer outcome in first-line therapy. These results were observed when HDC was used either as front-line treatment [19,27], or as consolidation therapy [17,28-32]. However published randomized phase III trials did not confirm these results. In a single center small-sized study from Papadimitriou *et al.*[19], although PFS was numerically improved by HDC (85.2 months versus 18 months), the difference was not significant (*p*=0.059). Moreover, no significant difference was observed in OS (not reached after 75 months of follow-up versus 75 months, p=0.38). The authors attributed PFS gain to the higher rates of stages IV (14% *vs.* 8.1%) and larger post-operative residue (32.6% *vs.* 21.6%) in the conventional therapy arm. Mobus *et al.* reported similar findings in their relatively large phase III trial published in 2007 [20]. Median PFS was 29.5 months in the HDC arm versus 20.5 in the control arm (p=0.40). There was also no difference regarding OS (54.4 *vs.* 62.8 months, *p*=0.54). Conclusions of these studies were that HDC does not improve outcome in advanced ovarian cancer.

Nevertheless a question that could be asked is: are these conclusions relevant for all patients or is there a subset of patients who may benefit from HDC? In this retrospective study, we tried to address this issue using a subgroup analysis approach in a large population of more than 160 patients. We have explored prognostic value of the different histoclinical features used in ovarian cancer evaluation: age, performance status, FIGO stage, histological subtype, histological grade, debulking status and response to conventional chemotherapy. Age was the only parameter correlated to HDC efficacy, both in PFS and OS. Intriguingly, patients under 50 years of age had a gain in survival when HDC was performed after platinum/taxane-based chemotherapy: median OS of 54.6 months *vs.* 36 months with standard treatment (*p*=0.05). This benefit was observed independently of the response after standard treatment. A possible hypothesis is that, in young patients known to have a better prognosis than older women, HDC may be more efficient regardless of the persistence of residual disease after conventional therapy. A hypothesis to explain these results could be the higher prevalence of *BRCA*-related tumors in younger patients compared to sporadic forms [33,34]. Indeed, *BRCA*-related ovarian cancers display distinctive biological and clinical characteristics including genomic instability, dysfunction in DNA repair processes especially homologous recombination and thereby higher sensitivity to platinum-based chemotherapy and better outcome [35,36]. Of note, recent data have shown that this phenotype could be extended to a larger group of tumors without germline *BRCA* mutations, the so-called “BRCAness” phenotype [37,38]. Thus, the benefit of alkylating agents-based HDC in younger patients observed in this study may reflect the enrichment in *BRCA*-related or BRCAness-associated forms in this subgroup and therefore a higher sensitivity of ovarian cancer cells to DNA damages that can be induced by alkylating agents. As suggested by the dose-effect concept, more chemotherapy –and thus more DNA lesions- may lead to an increase in tumor cells death.

A similar exploitation of this Achilles’ heel of the BRCAness-related phenotype was recently demonstrated with the new therapeutic class of PARP1 inhibitors [39], which also target DNA repair processes. PARP1 inhibitors are able to induce DNA single-strand breaks that will accumulate and degenerate to DNA double-strand breaks, which are not appropriately repaired if the BRCA pathway is deficient or dysfunctional, the so-called synthetic lethality concept. Olaparib has been shown to induce relevant and promising rates of response when used as single agent in AOC. Interestingly, its activity was documented not only in patients carrying *BRCA* mutations [40,41], but also in patients without constitutive mutations [42], further validating the BRCAness concept.

This phenomenon may be increased with the association of PARP inhibitor and alkylating drugs. Such an additive activity may not be necessary in case of complete remission after standard treatment, but may have a positive effect when the tumor burden has been decreased but not eliminated by the initial treatment.

Our observations show that more treatment may be more effective in young patients. Addition of HDC after platinum/taxane-based chemotherapy in this population should be compared to other ways to enhance treatment exposure. Intra-peritoneal chemotherapy may be an option to increase the doses of platinum and/or taxane administered to cancer cells, with less hematological adverse events [43]. Another issue is the lack of studies comparing consolidation (such as HDC) and maintenance therapy, which could be based on cytotoxic treatments [44] as well as angiogenesis inhibitors [45]. Nevertheless it is of note that, except angiogenesis inhibiting agents, none of the treatments cited above has shown his superiority in randomized trials versus observation alone, but without age consideration as we have done in this analysis. These new findings must be balanced with the fact that this study was retrospective, and that HDC regimens were heterogeneous. Nevertheless, despite its retrospective nature, this study, based on a large population, used a comparative design and included subgroup analyses with traditional clinical and pathological prognostic factors. Another limitation of this work is the absence of relevant information about the *BRCA* status of our patients. Unfortunately, this data was available only for few patients in our retrospective cohort (21 of 163), with only six *BRCA1* and two *BRCA2* mutations identified.

## Conclusions

We have shown in this retrospective comparative study including more than 160 women, that, when applied to all patients, HDC does not improve advanced ovarian cancer survival. However, HDC seems to benefit to young patients (less than 50 years of age). Median overall survival in this subset presented an improvement of 18 months when HDC was performed after initial platinum/taxane-based chemotherapy versus standard chemotherapy alone. This work is the first to make the hypothesis of a differential benefit from HDC according to age. As we know that young patients have a higher frequency of BRCA alterations than older women, they may have a more important benefit from HDC. That may lead to new clinical trials to explore this hypothesis of HDC usefulness in young patients, without or with combination with drugs targeting DNA repair such as olaparib.

## Abbrevations

AOC: Advanced ovarian carcinoma; CCA: Conventional chemotherapy alone; CCR: Clinical complete response; HDC: High-dose chemotherapy; HR: Hazard ratio; HSCS: Hematopoietic stem cell support; OS: Overall survival; PFS: Progression-free survival; SLS: Second look surgery.

## Competing interests

The authors declare that they have no competing interests.

## Authors’ contributions

Conception and design: RS. Acquisition of data: RS, AG, MAC, FR, EL, CC, PV, JME. Statistical analysis: RS. Manuscript writing: RS, AG, FB, JME. Final approval: all authors.

## Supplementary Material

Additional file 1 **Table S1.** Prognostic parameters (PFS) in stage IIIc patients, Cox regression analyses. Click here for file
